# Total IgE Trends in Children with Allergic Diseases

**DOI:** 10.3390/jcm13133990

**Published:** 2024-07-08

**Authors:** Nikolaos Katsanakis, Paraskevi Xepapadaki, Ioannis-Alexios Koumprentziotis, Pavlos Vidalis, John Lakoumentas, Maria Kritikou, Nikolaos G. Papadopoulos

**Affiliations:** Allergy Department, 2nd Pediatric Clinic, National and Kapodistrian University of Athens, 11527 Athens, Greece; nikoskatsanakis10@gmail.com (N.K.); vickyxepapadaki@gmail.com (P.X.); giannhskmpr@gmail.com (I.-A.K.); pavlosvid@hotmail.com (P.V.); john.lakoo@gmail.com (J.L.); nikpap@med.uoa.gr (N.G.P.)

**Keywords:** total IgE, immunoglobulin, biomarkers, allergy, children

## Abstract

**Background/Objectives:** The importance of non-invasive biomarkers for the diagnosis and monitoring of allergic diseases in childhood is currently unknown. From this perspective, data on the role of the total (t) immunoglobulin E (IgE) in relation to different allergic diseases across different age groups until adulthood remain unclear. The potential association of tIgE levels with types of allergic diseases diagnosed in an specialized tertiary allergy center, in relation to sex and the age group spanning from birth to 20 years, are evaluated in the present study. **Methods:** In this retrospective study, the tIgE values were obtained from children assessed for allergy-associated symptoms in our department from January 2015 to December 2020. The tIgE values were analyzed in relation to age and diagnosis. **Results:** Data from 2127 patients (1321 boys (62.1%)), with a median age of 6.31 (3.01–9.95) years, were available. The tIgE median values for the studied population were 132 (37.7–367.5) kU/lt. The tIgE values showed a significant increase from 0–2 years to 2–5 and 5–12 years, but not from 5–12 to 12–20 years. Boys exhibited significantly higher tIgE values compared to girls. Furthermore, the tIgE levels were significantly increased in children with asthma, allergic rhinitis, food allergy, and atopic dermatitis in comparison to children without these diagnoses. **Conclusions**: The total IgE values exhibit a significant and progressive longitudinal increase in children with allergic diseases, particularly notable in the 0–2 and 5–12 age groups, in boys, and in children diagnosed with atopic conditions.

## 1. Introduction

The worldwide prevalence of allergic diseases, especially immunoglobulin E (IgE)-mediated diseases, has increased dramatically in the last decades [1,2,3]. Asthma is one of the most prevalent chronic diseases globally, affecting approximately 350 million individuals [4]. The prevalence of food allergy in children varies depending on the diagnostic criteria employed, ranging from <4% to 7% according to various methodologies [5,6,7,8,9]. Data from Europe and the USA indicate a prevalence of 15–20% of atopic dermatitis (AD) in children [9], with an observed increase in the incidence in industrialized countries [10]. Similarly, data from five European countries show a point prevalence of 1.4% for chronic urticaria among the pediatric population [11], while significantly increased rates, up to 20% are observed for allergic reactions manifesting as acute urticaria [12].

According to the FDA-NIH, a biomarker is a defined measurable characteristic that serves as an indicator of a normal or pathophysiological biological process or response to an exposure or intervention [13]. Non-invasive biomarkers, such as the total (t) and specific immunoglobulin E (IgE), molecular allergens, blood or sputum eosinophils, and fractional exhaled nitric oxide (FeNO), have been proposed as predictive, diagnostic, and monitoring tools in children with allergic diseases [14,15,16,17].

While IgE is a key molecule in the allergic cascade, data on the utility of tIgE in the diagnosis and monitoring of allergic diseases are contradictory [18]. A systematic review and meta-analysis demonstrated that serum tIgE levels ≥ 60 kU/lt in children under 10 years of age are significantly linked to a heightened risk of developing asthma [19], while increased tIgE values are significantly associated with the diagnosis and severity of AD [20]. 

Nevertheless, data on the evolution of serum tIgE levels with age and their associations with different allergic diseases are limited. This study aims to assess the tIgE values across different age groups in children referred for suspected allergic diseases, from birth to early adulthood. Secondary outcomes include potential associations of tIgE values with sex and specific diagnoses, such as asthma, allergic rhinitis/conjunctivitis, drug-induced allergy, food allergy, hymenoptera allergy, AD, and urticaria, in a pediatric population derived from a specialized allergy center.

## 2. Materials and Methods

### 2.1. Patient Selection

We conducted a retrospective review of medical charts and laboratory data of individuals referred to the Allergy Department, 2nd Pediatric Clinic, National and Kapodistrian University of Athens, Greece due to suspected allergic symptoms between January 2015 and December 2020, and for whom tIgE measurements were available. The age range was 0–20 years. Children presented with a variety of symptoms suggestive of allergic diseases, such as chronic recurrent cough, persistent rhinitis, skin dryness, etc. It is worth noting that not all children presenting with allergy-related symptoms received a diagnosis of an allergic disease.

The diagnosis of allergic diseases, following current departmental practices and international recommendations, is performed during the initial clinical visit as follows: the diagnosis of asthma was based on a history of recurrent respiratory symptoms, such as wheeze, shortness of breath, chest tightness, and cough, with variable expiratory airflow limitation. This was assessed either by auscultation or positive bronchodilator reversibility testing for children over 5 years of age and markers of airway hyperresponsiveness, such as a cough during exercise. Preschool-aged children should have at least three wheezing episodes in the preceding 12 months (one in the last six months) and a positive response following anti-inflammatory treatment [21]. Allergic rhinitis diagnosis was based on recurrent episodes of nasal congestion, rhinorrhea (anterior and posterior), sneezing, and itching, associated with positive allergic sensitization [22]. Moreover, the diagnosis of food allergy required a definite history of IgE-mediated symptoms (i.e., immediate-type symptoms within 2 h following ingestion of the implicated food) and either a positive open food challenge (OFC) to the implicated food or a positive skin prick test (SPT)/specific IgE antibodies to the food greater than 0.35 kU/lt in the preceding 12 months. The diagnosis of atopic dermatitis was based on clinical criteria and a compatible history of atopic lesions, characterized by intense pruritus [23]. SIgE and skin prick tests were performed in all cases, either supporting the diagnosis in asthma and atopic dermatitis cases, or confirming the diagnosis in children with food allergies and allergic rhinitis.

Following the initial evaluation and respective allergy workup, some children did not meet the proposed criteria for an allergic disease or were eventually diagnosed with a different condition, such as an infectious disease (common cold, laryngitis, bronchitis, bronchiolitis), or other chronic diseases. Based on their evaluation, the children were further categorized based on whether they had zero, one, or more than one allergic disease. The children included in the study were not on biological agents in order to avoid alterations in IgE values and clinical presentation of the disease.

The data collected and assessed included gender, age, date of evaluation and examination, tIgE values, and the diagnosis of allergic disease. In this study, we categorized the children into four distinct age groups at the initial evaluation: 0–2 years, 2–5 years, 5–12 years, and 12–20 years. To avoid overlap, each group’s beginning was set on the individual’s birth date and ended the day before their next birthday. This stratification allowed for a more precise analysis of how allergic conditions manifest and progress across different developmental stages. The data analyzed were derived from the children’s files for whom parental consent was obtained during the initial visit for anonymous data analysis, following the established practice in our department. The Ethics Committee of the P-A Kyriakou Children’s Hospital approved the obtaining and analysis of the data from files with parental consent.

### 2.2. Statistical Analysis

All scale variables, such as IgE (kU/lt) and age (years), were assessed for normality using the Shapiro–Wilk test, revealing non-normal distributions. Subsequently, non-parametric descriptive and inferential statistics were applied. Descriptive statistics are presented as the median (Q1–Q3) for scale variables, and as the count (%) for categorical variables. 

Potential associations between IgE and the presence or absence of any disease’s status were examined using the Wilcoxon’s rank-sum test. Correlation analysis between IgE and age was conducted with Spearman’s rho, and their optimal relationship was assessed with a polynomial test. Polynomial degrees ranging from 1 (linear) to 10 were assessed with the optimal degree selected, based on the maximized of the adjusted R squared metric. In cases where the IgE levels were compared among multiple groups (more than two), the Kruskal–Wallis test was applied, combined with Dunn’s test and the Bonferroni correction method to account for post-hoc pairwise comparisons.

All statistical tests were considered two-sided, and statistical significance was set at the 5% level. Statistical analysis and visualization were conducted using R language for statistical computing and RStudio IDE, both of which are open-source products. IgE values equal to or greater than 2000 kU/lt were recorded as ‘’2000 kU/lt’’ due to the maximum measurement capacity of the diagnostic tools utilized. 

## 3. Results

### 3.1. Population Demographic Characteristics

The initial population of the study comprised 2324 children, with complete data for inclusion and analysis for 2127 children. These children accounted for 91.5% of the initial population, had a median age of 6.31 years (3.01–9.95), and 1321 were boys (62.1%). The baseline characteristics of the entire included population are presented in Table 1. 

The number of children, median age, sex, and respective tIgE values in each age group were as follows: 0–2 years: 362 children, median age of 1.06 years (IQR: 0.68–1.44), 197 boys (54.4%), with tIgE values of 42.6 kU/lt (IQR: 14.2–139.7); 2–5 years: 482 children, median age of 3.5 years (IQR: 2.66–4.29), 289 boys (59.9%), with tIgE values of 88.5 kU/lt (IQR: 32.9–260.5); 5–12 years: 1003 children, median age of 8.3 years (IQR: 6.55–9.97), 641 boys (63.9%), with tIgE values of 189 kU/lt (IQR: 57.3–456); 12–20 years: 280 children, median age of 13.74 years (IQR: 12.82–15.17), 194 boys (69.2%), with tIgE values of 232 kU/lt (IQR: 98.3–464.5).

### 3.2. tIgE Values in the Different Age Groups

**Figure 1:**
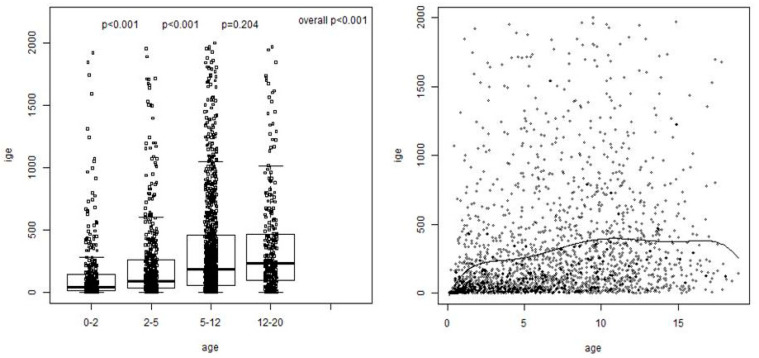
Median tIgE (in kU/lt) in each age group, showing statistical correlations (**left**), and correlation of tIgE values for the entire population—polynomial degree of 6 (**right**).

The median value of the tIgE for the whole cohort was 132 (37.7–367.5) kU/lt (Table 1). Significant increases in tIgE values were observed between the 0–2 and the 2–5 age groups (from 42.6 (14.2–139.7) kU/lt to 88.5 (32.9–260.5) kU/lt, *p* < 0.001) and the 2–5 and 5–12 age groups (from 88.5 (32.9–260.5) kU/lt to 189 (57.3–456) kU/lt, *p* < 0.001). Alterations in the tIgE values between the 5–12 and 12–20 age groups did not reach statistical significance (*p* = 0.204) (Figure 1).

A significant positive correlation was noted between the tIgE levels and the patients’ ages for the entire cohort (rho = 0.33, *p* < 0.001), and specifically within the 0–2 age group (rho = 0.42) and the 5–12 age group (rho = 0.15) (*p* < 0.001) (Figure 1).

### 3.3. Correlation between tIgE Values and the Number of Allergic Diseases

The number of children with a documented diagnosis of an allergic disease, including asthma, allergic rhinitis, food allergy, atopic dermatitis, drug- or hymenoptera-induced allergy, allergic conjunctivitis, and urticaria, and the numbers of specific diagnoses and their respective tIgE values were determined both overall and within each age group separately (the data for each diagnosis across the entire population and within each age group are shown in Table 2). Additionally, the tIgE values associated with each diagnosis in the entire population and within each age group are presented in Table 3. Moreover, the data comparing the tIgE values in children without any diagnosed allergic disease versus those with one (or more) diagnoses are summarized in Table 4.

Out of the 2127 children, 836 (39.3%) did not receive a diagnosis of an allergic disease, while 919 (43.2%) were diagnosed with one, and 372 (17.5%) with more than one allergic disease after the thorough allergy evaluation (Table 4). In total, 1758 diagnoses were made for the 1291 children who were diagnosed with at least one condition. The distribution of the diagnoses was as follows: asthma—455 (25.9%), allergic rhinitis—482 (27.4%), food allergy—417 (23.7%), drug allergy—28 (1.6%), atopic dermatitis—156 (8.9%), hymenoptera allergy—4 (0.2%), allergic conjunctivitis—23 (1.3%), other allergies—142 (8.1%), urticaria—51 (2.9%).

Regarding the number of diagnoses, increased values of tIgE were observed in children with more than one allergic disease compared to those without any or with only one allergic disease (*p* < 0.001). This trend was also observed within each age group with the 0–2, 5–12, and 12–20 age groups being statistically significant (*p* = 0.016, *p* < 0.001, *p* = 0.013, respectively), while for the 2–5 age group, the correlation was marginal (*p* = 0.057).

### 3.4. Comparisons of tIgE Values between Children with and without an Allergic Disease

Comparisons of the tIgE values were performed between children with and without a documented allergic disease. Specifically, the tIgE was significantly increased in children with asthma (compared to those without): 172 kU/lt, IQR: 50–383.5 kU/lt vs. 123 kU/lt, IQR: 36.1–359.2 kU/lt (*p* = 0.003); allergic rhinitis: 188 kU/lt, IQR: 50.9–418 kU/lt vs. 120 kU/lt, IQR: 36.2–351 kU/lt (*p* < 0.001); food allergy: 208 kU/lt, IQR: 51–463 kU/lt vs. 118 kU/lt, IQR: 36.3–341.7 kU/lt (*p* < 0.001); and atopic dermatitis: 219 kU/lt, IQR: 70.5–515.5 kU/lt vs. 125 kU/lt, IQR: 36.3–356.5 kU/lt (*p* = 0.001). Comparisons for drug/hymenoptera allergy, urticaria, and allergic conjunctivitis did not yield significant differences (Supplementary Table S1). 

### 3.5. Comparisons of tIgE Values between Children with Specific Diseases in the Different Age Groups

In the 0–2 age group, significant tIgE differences were noted for atopic dermatitis compared to children with no allergies (72.7 kU/lt (IQR: 45.2–202 kU/lt) vs. 37.8 kU/lt (IQR: 13.6–133 kU/lt), *p* = 0.012), while in the 2–5 age group, increased tIgE values were observed in children with food allergy compared to those without (171 kU/lt (IQR: 85.1–402 kU/lt) vs. 71.8 kU/lt (IQR: 29.1–214.5 kU/lt), *p* < 0.001). Moreover, in the 5–12 age group, elevated tIgE values were observed in children with food allergy (343 kU/lt (IQR: 165.5–709.7 kU/lt) vs. 160 kU/lt (IQR: 48–418 kU/lt), *p* < 0.001) and atopic dermatitis (259 kU/lt (IQR: 137.7–518.5 kU/lt) vs. 176 kU/lt (IQR: 53.4–446 kU/lt), *p* = 0.03). In line, in the 12–20 age group, tIgE values were increased in children with documented food allergy (362 kU/lt (IQR: 217–664 kU/lt) vs. 206 kU/lt (IQR: 78.5–428 kU/lt), *p* < 0.001) and atopic dermatitis (529.5 kU/lt (IQR: 227.5–814.2 kU/lt) vs. 216 kU/lt (IQR: 94.1–453 kU/lt), *p* = 0.001).

### 3.6. tIgE Values and Sex

The tIgE values were significantly higher in boys: 162 (46.4–426.2) kU/lt, compared to girls: 91.5 (26.7–255.5) kU/lt, *p* < 0.001 for the whole cohort. This predominance of tIgE values in boys with allergic disease was pronounced in the 0–2 age group: 54.7 (18.2–170) kU/lt vs. 31 (11.2–102) kU/lt, *p* = 0.02; the 2–5 age group: 110.5 (34.6–297.7) kU/lt vs. 68.5 (28–186) kU/lt, *p* = 0.08; and the 5–12 age group: 233 (85.4–544.5) kU/lt vs. 119 (39.5–323.7) kU/lt, *p* < 0.01. 

No difference was observed in the total IgE values between boys and girls in the 12–20 age group: 240 (94.9–468) kU/lt vs. 216 (119.2–466.7) kU/lt, *p* = 0.78. 

## 4. Discussion

In this retrospective study, we observed age-related changes in the tIgE values across the pediatric life span, as well as in relation to specific allergic diseases within predefined age groups. It is worth noting that there was a significant increase from birth to the age of 2 years and from 5 to 12 years. Moreover, correlations between the tIgE and the diagnoses of asthma, allergic rhinitis, food allergy, and atopic dermatitis diagnosis were observed. Stronger associations of the tIgE in the 0–2, 5–12, and 12–20 age groups were observed with atopic dermatitis, followed by food allergy, allergic rhinitis, and asthma. These findings underscore the importance of considering age-specific patterns of tIgE elevation and its associations with various allergic diseases in pediatric populations.

Consistent with our findings, recent reports have suggested the significance of persistent high tIgE values (≥ 200 kU/lt) from infancy to early childhood as a risk factor for the development of AD, allergic rhinitis, and asthma in preschool years [24]. Moreover, Sacco et al. proposed that consistently increased tIgE quantiles predict atopic sensitization by the age of 5 years [25], although this has not been always confirmed [26]. Furthermore, a study from Taiwan that included children with and without atopic predisposition demonstrated a diminished diagnostic accuracy of tIgE levels with respect to allergic diseases’ diagnoses, irrespective of the cutoff values. This implies that the tIgE might be more strongly associated with atopy than the symptoms per se [27]. In our study, the tIgE values were strongly associated with the diagnosis of an allergic disease, suggesting that tIgE values could potentially serve as a confirmatory diagnostic biomarker. In accordance, Matricardi et al. demonstrated that increased tIgE values or longitudinal increases over time in previously healthy children predicted the occurrence of allergic diseases in children [28].

While our study was not specifically designed for this analysis, we observed elevated tIgE values in children with multiple allergic morbidities, consistent with previous reports indicating higher tIgE levels in children sensitized to multiple allergens [29]. Additionally, polysymptomatic pediatric patients, compared to monosymptomatic or asymptomatic cases, exhibited increased tIgE values [30]. Furthermore, recent findings demonstrated that polysensitized patients with allergic rhinitis displayed higher ocular, itch-related, and total rhinitis symptom scores compared to mono-sensitized individuals [31], while children diagnosed with both atopic dermatitis and asthma had higher tIgE values compared to those with either disease alone [32].

Another finding in our study was increased tIgE values in boys with allergic disease, from birth to pre-adolescence, in accordance with the known male predominance of allergic diseases during childhood [33,34]. This difference might be because hormonal influences, environmental and social factors, or sex-specific genetic predisposition cease to exist in adolescence [1]. In our study, we found differences in the tIgE values among different age groups, which represent distinct maturation and disease presentation landmarks. Moreover, we found differences between children with one or multiple allergic diseases with respect to their total IgE values, further supporting the notion that atopic sensitization is strongly associated and potentially contributes to allergic morbidity in a quantitative manner.

We also noted an increased number of diagnosed allergic diseases in the 12–20-year old age group. This finding may be evident in this age group because, as is known, in atopic children, additional clinical syndromes may manifest over time and fully evolve at older ages to be formally diagnosed as allergic diseases.

Limited data exist on the longitudinal aspects of tIgE measurements in patients with allergic diseases. Several studies highlight the importance of tIgE as both a diagnostic tool and a marker of severity in children with atopic dermatitis [35,36], while Somani et al. reported that peak tIgE values in these children can be observed between 10 and 20 years of age [37]. In addition, the risk of presenting asthma and food allergy has been significantly correlated with tIgE values, although individual variations may limit its diagnostic accuracy [38].

Omalizumab, a monoclonal antibody that binds to IgE, is currently approved for severe asthma and is used both on- and off-label for various allergic diseases, including chronic spontaneous urticaria, rhinosinusitis with polyps, and severe food allergies. Although recommendations limit the use of anti-IgE therapy to patients with severe asthma and IgE levels not exceeding 1500 KU/L, significant beneficial effects have been observed in patients with severe asthma and food allergies with IgE levels greater than 1500 KU/L, in terms of asthma control and food desensitization to the offending foods [39]. Moreover, it is well established that high tIgE levels predict a favorable response to omalizumab in antihistamine-refractory chronic urticaria cases [40]. These data underpin the significant role of assessing tIgE levels with respect to therapeutic approaches.

The Allergy Department of the 2nd Pediatric Clinic in Athens is the largest referral center for children with food allergies in Greece. Therefore, the relatively high frequency of food-allergic children in our center cannot be extrapolated to the general population. In contrast, previous studies have shown that the prevalence of food allergies in children in Greece is relatively low compared to other parts of Europe [8,41]. Moreover, due to hot and humid weather conditions, there is a relatively low rate of children with atopic dermatitis, and severe cases are even less pronounced [42].

The main limitations of our study include its retrospective design and the assumption that children without a diagnosis of allergic disease at the time of assessment did not have the disease. Furthermore, the sample size of children with certain allergies, such as drug- or hymenoptera-related allergy, urticaria, and conjunctivitis, was too small to draw firm conclusions. Another weakness was that the laboratory evaluation including tIgE values was performed at the time of the allergist’s evaluation, and a significant amount of time may have elapsed since the original referral, particularly for non-emergency cases. As a result, our data are correlated with the disease diagnosis but not necessarily with disease activity at the time of evaluation. Moreover, reverse causation, meaning that high IgE values could potentially enhance the diagnosis of allergic diseases cannot be excluded. Nevertheless, the study’s major strengths include the high number of children included in the analysis and the thorough evaluation by allergy specialists for each diagnosis, providing robustness to our findings.

## 5. Conclusions

In conclusion, our findings highlight that the total tIgE levels exhibit a progressive increase over time, particularly notable in the 0–2 and 5–12 age groups, and in children diagnosed with allergic diseases. Moreover, elevated tIgE values were consistently observed in males, in cases of multi-allergic conditions, and in children diagnosed with atopic conditions (asthma, allergic rhinitis, food allergy, and atopic dermatitis). Our study suggests that tIgE values could serve as an additional biomarker to support the diagnosis of allergic diseases, also considering the age of the patient. These findings underscore the potential utility of tIgE assessment in clinical practice for the management and diagnosis of allergic conditions.

## Figures and Tables

**Table 1:** Number of males, median (IQR) age, and median (IQR) tIgE (kU/lt) in the whole cohort and in each age group.

	All Children (n = 2127)	0–2 Years (n = 362)	2–5 Years (n = 482)	5–12 Years (n = 1003)	12–20 Years (n = 280)
Gender, male, n (%)	1321 (62.1%)	197 (54.4%)	289 (59.9%)	641 (63.9%)	194 (69.2%)
Age in years (median, IQR)	6.31 (3.01–9.95)	1.06 (0.68–1.44)	3.5 (2.66–4.29)	8.3 (6.55–9.97)	13.74 (12.82–15.17)
Total IgE (median, IQR)	132 (37.7–367.5)	42.6 (14.2–139.7)	88.5 (32.9–260.5)	189 (57.3–456)	232 (98.3–464.5)

**Table 2:** Diagnoses of allergic diseases for the whole cohort and for each age group.

	All Diagnoses (n = 1758)	0–2 Years (n = 187)	2–5 Years (n = 336)	5–12 Years (n = 931)	12–20 Years (n = 304)
Asthma, n (%)	455 (25.9%)	19 (10.2%)	99 (29.4%)	255 (27.4%)	82 (27%)
Allergic Rhinitis, n (%)	482 (27.4%)	6 (3.2%)	54 (16.1%)	307 (33%)	115 (37.8%)
Food Allergy, n (%)	417 (23.7%)	120 (64.2%)	102 (30.4%)	142 (15.3%)	53 (17.4%)
Drug Allergy, n (%)	28 (1.6%)	1 (0.5%)	6 (1.8%)	16 (1.7%)	5 (1.6%)
Atopic Dermatitis, n (%)	156 (8.9%)	29 (15.5%)	35 (10.4%)	74 (7.9%)	18 (5.9%)
Hymenoptera Allergy, n (%)	4 (0.2%)	1 (0.5%)	1 (0.3%)	1 (0.1%)	1 (0.3%)
Urticaria, n (%)	51 (2.9%)	1 (0.5%)	8 (2.4%)	37 (4%)	5 (1.7%)
Other Allergies, n (%)	142 (8.1%)	10 (5.4%)	29 (8.6%)	84 (9%)	19 (6.3%)

**Table 3:** Total IgE (kU/lt) values of children with an allergic disease for the entire population and for each age group.

	All Children (n = 2127)	0–2 Years (n = 362)	2–5 Years (n = 482)	5–12 Years (n = 1003)	12–20 Years (n = 280)
	IgE (median, IQR)	IgE (median, IQR)	IgE (median, IQR)	IgE (median, IQR)	IgE (median, IQR)
Asthma	172 (50–383.5)	80.7 (15.8–188)	68.5 (27.7–160.5)	218 (69.2–450.5)	242 (135.2–467.5)
Allergic Rhinitis	188 (50.9–418)	105.8 (83–136.2)	37.3 (15.9–136.5)	214 (57.3–447.5)	240 (112–446.5)
Food Allergy	208 (51–463)	39.9 (11.2–161)	171 (85.1–402)	343 (165.5–709.7)	362 (217–664)
Drug Allergy	137.1 (39.9–351)	429 (429–429)	74.9 (34.7–245.9)	123.9 (39.2–277.7)	429 (94.2–558)
Atopic Dermatitis	219 (70.5–515.5)	72.7 (45.2–202)	92.2 (42.6–392.5)	259 (137.7–518.5)	529.5 (227.5–814.2)
Hymenoptera Allergy	113.6 (78.9–162.7)	243 (243–243)	91.3 (91.3–91.3)	41.9 (41.9–41.9)	136 (136–136)
Urticaria	180 (49–335)	1.5 (1.5–1.5)	146 (59.9–272.2)	198 (50.4–357)	159 (91.7–169)
Other Allergies	109.5 (35.2–359)	43.95 (26.7–190.5)	41.6 (28–119)	153.5 (42.8–449.5)	217 (46.5–456)

**Table 4:** Number of children and comparison of median (IQR) tIgE (kU/lt) values in those without documented/diagnosed allergic disease vs. those with one vs. those with more than one. * *p* values correspond to IgE differences in children being diagnosed with 0–1 or more allergic diseases in each age group respectively and in total).

	All Children (n = 2127)	0–2 Years (n = 362)	2–5 Years (n = 482)	5–12 Years (n = 1003)	12–20 Years (n = 280)
*Number of diagnoses (percentage of the corresponding total)*					
0 Allergies	836 (39.3%)	197 (54.4%)	217 (45%)	344 (34.3%)	78 (27.9%)
1 Allergy	919 (43.2%)	147 (40.6%)	207 (43%)	444 (44.3%)	121 (43.2%)
>1 Allergies	372 (17.5%)	18 (5%)	58 (12%)	215 (21.4%)	81 (28.9%)
*Total IgE values, ku/lt (IQR)*					
0 Allergies	112.5 (36.1–326.5)	37.8 (15.3 102)	87.4 (36.8–290)	157.5 (68.9–513)	203.5 (80.1–446.7)
1 Allergy	116 (32–320)	40.9 (12.3–163.5)	76.6 (28.8–218.5)	145 (41.9–370)	230 (64.8–453)
>1 Allergies	257.5 (104.2–515.5)	121 (74.4–246.2)	125.5 (39.3–348.7)	292 (111–574.5)	309 (146–657)
*p* value * (overall)	**<0.01**	**0.016**	0.057	**<0.01**	**0.013**

## Data Availability

The data that support the findings of this study are available from the corresponding author upon reasonable request.

## Supplementary Materials

The following supporting information can be downloaded at: https://www.mdpi.com/article/10.3390/jcm13133990/s1, Table S1: Total immunoglobulin E (TIgE) values for each disease, which are compared with all the other children without that specific diagnosed disease. Data are presented for the whole cohort and for the different age groups. Data regarding the differences in female and male patients are presented here.
