# Supplementary material for: Total IgE Trends in Children with Allergic Diseases
Source: J Clin Med. 2024 Jul 8;13(13):3990. doi: 10.3390/jcm13133990 (PMC11242130; doi:10.3390/jcm13133990)
Supplement: Supplementary file 1 [file jcm-13-03990-s001.zip › jcm-3046463-supplementary.pdf]

Supplementary Table S1. Total immunoglobulin E (TIgE) values for each disease, which are compared with all the other children without that specific diagnosed disease. Data are presented for the whole cohort and for the different age groups. Data regarding the differences in female and male patients are presented here.

**ALL COHORT**	**GENDER (MALE/FEMALE)**	162 (46.45 - 426.25)	91.5 (26.75 - 255.5)	**p<0.001**
	**AS (NO/YES)**	123 (36.175 -359.25)	172 (50 - 383.5)	**p=0.003**
	**AR (NO/YES)**	120 (36.2 - 351)	188 (50.9 - 418)	**p<0.001**
	**FA (NO/YES)**	118 (36.325 -341.75)	208 (51 - 463)	**p<0.001**
	**DA (NO/YES)**	132 (37.7 - 369)	137.1 (39.925 - 351)	p=0.988
	**AD (NO/YES)**	125 (36.3 - 356.5)	219 (70.575 - 515.5)	**p=0.001**
	**HY (NO/YES)**	132 (37.65 - 369)	113.65 (78.95 -162.75)	p=0.778
	**OT (NO/YES)**	134 (37.8 - 368)	109.5 (35.25 - 359)	p=0.605
	**UR (NO/YES)**	131.3 (37.6 - 371)	180 (49.05 - 335)	p=0.659
	**CO (NO/YES)**	131.8 (37.6 - 363.25)	217 (84.9 - 527)	p=0.075
**0-2 YEARS OLD**	**GENDER (MALE/FEMALE)**	54.75 (18.25 - 170)	31 (11.2 - 102)	**p=0.002**
	**AS (NO/YES)**	42.2 (14.15 - 123)	80.7 (15.8 - 188)	p=0.418
	**AR (NO/YES)**	41.15 (14.1 - 135.25)	105.8 (83.075 - 136.25)	p=0.054
	**FA (NO/YES)**	43.1 (15.825 - 113.75)	39.95 (11.2 - 161)	p=0.983
	**DA (NO/YES)**	42.6 (14.2 - 133)	429 (429 - 429)	p=0.154
	**AD (NO/YES)**	37.8 (13.6 - 133)	72.7 (45.2 - 202)	**p=0.012**
	**HY (NO/YES)**	42.6 (14.2 - 133)	243 (243 - 243)	p=0.232
	**OT (NO/YES)**	42.4 (14.1 - 135.25)	43.95 (26.725 - 190.5)	p=0.526
	**UR (NO/YES)**	42.6 (14.4 - 142)	1.5 (1.5 - 1.5)	p=0.104
	**CO (NO/YES)**	42.6 (14.25 - 139.75)	-	-
**2-5 YEARS OLD**	**GENDER (MALE/FEMALE)**	110.5 (34.65 - 297.75)	68.5 (28 - 186)	**p=0.008**
	**AS (NO/YES)**	94.1 (34.15 - 277)	68.5 (27.7 - 160.5)	p=0.056
	**AR (NO/YES)**	92.2 (36.15 - 279)	37.35 (15.9 - 136.5)	**p=0.001**
	**FA (NO/YES)**	71.85 (29.15 - 214.5)	171 (85.15 - 402)	**p<0.001**
	**DA (NO/YES)**	89.05 (33.05 - 259.5)	74.95 (34.725 - 245.975)	p=0.769
	**AD (NO/YES)**	87.4 (32 - 249.5)	92.2 (42.65 - 392.5)	p=0.422
	**HY (NO/YES)**	88.2 (32.9 - 261)	91.3 (91.3 - 91.3)	p=0.98
	**OT (NO/YES)**	92.2 (33.6 - 264)	41.6 (28 - 119)	p=0.073
	**UR (NO/YES)**	87.8 (32.375 - 257)	146.05 (59.925 - 272.25)	p=0.628
	**CO (NO/YES)**	87.8 (32.725 - 259.5)	308.5 (208.25 - 408.75)	p=0.297
**5-12 YEARS OLD**	**GENDER (MALE/FEMALE)**	233 (85.4 - 544.5)	119 (39.525 - 323.75)	**p<0.001**
	**AS (NO/YES)**	176 (51.65 - 460.75)	218 (69.25 - 450.5)	p=0.509
	**AR (NO/YES)**	175.5 (58.05 - 465.5)	214 (57.3 - 447.5)	p=0.975
	**FA (NO/YES)**	160 (48 - 418)	343 (165.5 - 709.75)	**p<0.001**
	**DA (NO/YES)**	190 (58.2 - 461.5)	123.95 (39.225 - 277.75)	p=0.202
	**AD (NO/YES)**	176 (53.4 - 446)	259 (137.75 - 518.5)	p=0.035
	**HY (NO/YES)**	189.5 (57.575 - 456.5)	41.9 (41.9 - 41.9)	p=0.301
	**OT (NO/YES)**	190 (59 - 458.5)	153.5 (42.825 - 449.5)	p=0.383
	**UR (NO/YES)**	188 (57.575 - 466.5)	198 (50.4 - 357)	p=0.703
	**CO (NO/YES)**	187.5 (57.275 - 452.75)	217 (95.1 - 547.5)	p=0.425
**12-20 YEARS OLD**	**GENDER (MALE/FEMALE)**	240 (94.9 - 468)	216 (119.25 - 466.75)	p=0.788
	**AS (NO/YES)**	215 (90.425 - 460)	242 (135.25 - 467.5)	p=0.153
	**AR (NO/YES)**	230 (90 - 488)	240 (112 - 446.5)	p=0.832
	**FA (NO/YES)**	206 (78.55 - 428)	362 (217 - 664)	**p<0.001**
	**DA (NO/YES)**	230 (100.75 - 461.5)	429 (94.2 - 558)	p=0.616
	**AD (NO/YES)**	216 (94.125 - 453)	529.5 (227.5 - 814.25)	**p=0.001**
	**HY (NO/YES)**	234 (98.15 - 466)	136 (136 - 136)	p=0.569
	**OT (NO/YES)**	234 (103 - 463)	217 (46.55 - 456)	p=0.528
	**UR (NO/YES)**	235 (100.75 - 469.5)	159 (91.7 - 169)	p=0.264
	**CO (NO/YES)**	232 (103.25 - 462.25)	177.55 (80.3 - 473.25)	p=0.762

Abbreviations: asthma AS; allergic rhinitis AR; food allergy FA; drug allergy DA; atopic dermatitis AD; hymenoptera related allergy HY; other allergies OA; urticaria UR; conjunctivitis CO.
